# Multiple Metastases of the Liver and Lung After Breast-Conserving Surgery for Ductal Carcinoma *In Situ* Without Microinvasion of the Breast: A Case Report and Literature Review

**DOI:** 10.3389/fonc.2022.855899

**Published:** 2022-04-11

**Authors:** Zhen Wang, Xinyang Zhang, Huiyang Ren, Lei Zhang, Bo Chen

**Affiliations:** Department of Breast Surgery, The First Hospital of China Medical University, Shenyang, China

**Keywords:** ductal carcinoma *in situ*, distant metastases, breast cancer, microinvasion, breast-conserving surgery

## Abstract

**Background:**

Ductal carcinoma *in situ* (DCIS) is a non-invasive disease that rarely causes distant metastasis. It is extremely rare for patients diagnosed with DCIS without microinvasion to develop distant metastasis in the absence of ipsilateral or contralateral breast recurrence. This is the first case report of multiple liver and lung metastases from DCIS after breast-conserving surgery and radiotherapy.

**Case Presentation:**

A 45-year-old woman who was diagnosed with DCIS and received breast-conserving surgery, radiotherapy, and sequential endocrine therapy developed multiple metastases in the liver and lung despite not having bilateral breast recurrence at the 62-month follow-up. Comprehensive advanced breast cancer therapy was administered but did not prevent the progression of metastatic foci in the liver.

**Conclusions:**

This case shows the poor potential outcome in DCIS. Further research should be conducted on metastasis in DCIS; reexamination and monitoring are indispensable for patients diagnosed with DCIS.

## 1 Introduction

Ductal carcinoma *in situ* is a non-invasive disease. It has a rare potential to cause distant metastasis (DM), which has been reported at a rate of 0.14% among 2,123 patients diagnosed with ductal carcinoma *in situ* (DCIS) (1). The COBCG-01 study collected the clinical data of 1,072 women diagnosed with DCIS treated with breast conservation surgery (BCS) and radiotherapy; four subsequent metastases occurred but only after invasive local recurrence (2). Therefore, DM without invasive ipsilateral or contralateral recurrence after performing BCS to remove the primary tumor is rare. Moreover, it is extremely uncommon for a woman diagnosed with DCIS without microinvasion to discover multiple DMs that occurred in more than one organ as the first event.

Here, we report a case of multiple DMs in the liver and lung as the first event after BCS and radiotherapy for DCIS without microinvasion of the breast and provide a literature review to explore the underlying reasons and formulate logical conclusions.

## 2 Case Presentation

### 2.1 Surgical Treatment of the Primary Tumor

**Figure 1:**
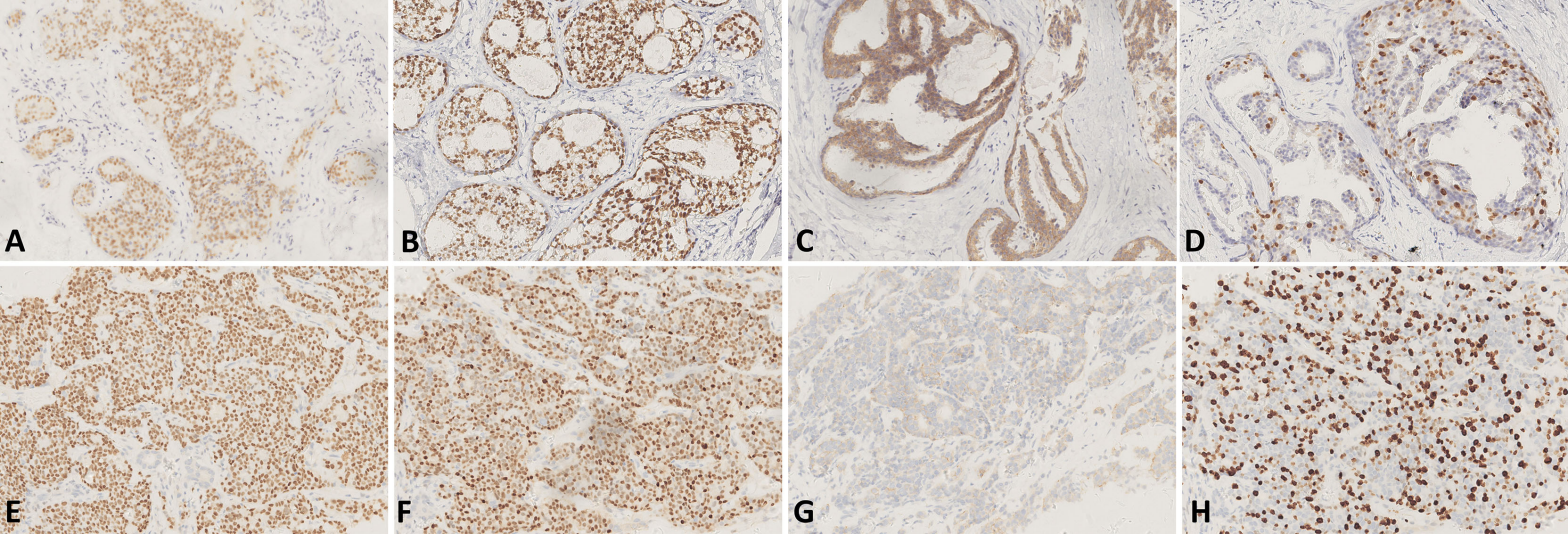
**(A–D)** IHC of the tissue of excisional biopsy of the right breast in 2012 [×200, **(A)** IHC for ER, **(B)** IHC for PR, **(C)** IHC for HER2, and **(D)** IHC for Ki-67]. **(E–H)** IHC of the tissue of liver biopsy in 2017 [×200, **(E)** IHC for ER, **(F)** IHC for PR, **(G)** IHC for HER2, and **(H)** IHC for Ki-67].

In 2012, a 45-year-old woman complained of right nipple discharge (white, occasionally streaked with blood). Imaging tests revealed a tumor (2.0 × 1.5 × 0.5 cm) located in the outer upper quadrant of the right breast. An excisional biopsy was performed at the First Hospital of China Medical University on 5 April 2012. The findings revealed intraductal papilloma, moderate-to-severe atypical hyperplasia, and malignant transformation of the local ductal epithelium. Immunohistochemistry (IHC) detected estrogen receptor (ER) (35%+), progesterone receptor (PR) (75%+), human epidermal growth factor receptor 2 (HER2) (intraduct: 3+), and Ki-67 (25%+) (**Figures 1A–D**). On 18 April 2012, BCS and sentinel lymph node biopsy were performed at the same hospital. An elliptical incision was created in the upper outer quadrant of the right breast, and quadrantectomy with posterior areolar gland resection was performed. Five safety margins were obtained and submitted for intraoperative frozen tissue pathology. No cancers were detected. An arc incision was made in the right axillary region, and two lymph nodes were sent for pathology. The postoperative pathological diagnosis revealed no lymph node involvement. The breast cancer was graded as pTisN0M0.

#### 2.1.1 Adjuvant Treatment After Surgery and Detection of Recurrence

**Figure 2:**
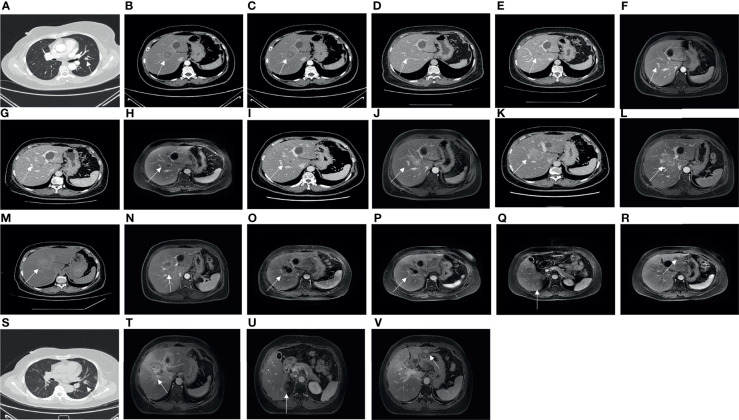
On 2 May 2017, CT showed a pulmonary metastasis with a long diameter of 1.3 cm **(A)**. On 31 May 2017, CT showed a hepatic metastasis with a long diameter of 4.7 cm on the right anterior lobe **(B)**. On 30 October 2017, CT showed a hepatic metastasis with a long diameter of 2.0 cm before involved in a clinical trial of adjuvant endocrine therapy on the right anterior lobe **(C)**. On 30 August 2019, CT showed no apparent hepatic metastasis **(D)**. On 21 November 2019, CT showed a hepatic metastasis with a long diameter of 1.8 cm on the right anterior lobe (unclear boundary) **(E)**. On 25 November 2019, MRI showed a hepatic metastasis with a long diameter of 1.2 cm on the right anterior lobe **(F)**. On 15 June 2020, CT showed a hepatic metastasis with a long diameter of 1.5 cm on the right anterior lobe **(G)**, and MRI showed hepatic metastases, especially the biggest one with a long diameter of 2.7 cm on the right anterior lobe **(H)**. On 11 December 2020, CT showed a hepatic metastasis with a long diameter of 3.2 cm on the right anterior lobe **(I)**, and MRI showed a hepatic metastasis with a long diameter of 3.6 cm on the right anterior lobe **(J)**. On 26 February 2021, CT showed a hepatic metastasis with a long diameter of 3.2 cm on the right anterior lobe **(K)**, and MRI showed a hepatic metastasis with a long diameter of 4.4 cm on the right anterior lobe **(L)**. On 21 April 2021, CT showed a hepatic metastasis with a long diameter of 5.6 cm on the right anterior lobe **(M)**, and MRI showed a hepatic metastasis with a long diameter of 6.0 cm on the right anterior lobe **(N)**. On 6 August 2021, MRI showed a hepatic metastasis with a long diameter of 5.0 cm on the right anterior lobe **(O)**. On 9 October 2021, MRI showed a hepatic metastasis with a long diameter of 3.5 cm on the right anterior lobe **(P)**, a hepatic metastasis with a long diameter of 1.0 cm on the right posterior lobe **(Q)**, and a hepatic metastasis with a long diameter of 1 cm on the left lateral lobe **(R)**. On 21 October 2021, the last CT showed a pulmonary metastasis with a long diameter of 1.0 cm **(S)**. On 15 December 2021, MRI showed a hepatic metastasis with a long diameter of 6.7 cm on the right anterior lobe **(T)**, a hepatic metastasis with a long diameter of 1.0 cm on the right posterior lobe **(U)**, and a hepatic metastasis with a long diameter of 1 cm on the left lateral lobe **(V)**.

Adjuvant radiotherapy was administered at the same hospital from May to June 2012. The dose was one course of treatment: DT50Gy. Concurrently, sequential tamoxifen endocrine therapy (20 mg/day) was administered until March 2017 (4 years and 10 months). After the surgery, regular examinations were conducted. Breast and liver ultrasounds were performed every 3 months. Mammography of the breast and computed tomography (CT) of the lung were performed annually. No recurrence or metastasis was observed until May of 2017. CT of the lung revealed multiple nodules in both lungs (**Figure 2A**), and abdominal ultrasound exhibited space-occupying lesions in the right anterior and posterior segments of the liver, which were confirmed by CT (**Figure 2B**). However, imaging examination revealed no recurrence in the bilateral breast and no lymph node metastasis. The pathological examination after liver biopsy on 7 June 2017, confirmed metastatic breast carcinoma; IHC detected ER (90%+), PR (75%+), HER2 (1+), Ki-67 (about 30%+), FISH (non-amplification) (**Figures 1E–H**), CK7 (+), E-cadherin (+), GATA-3 (+), hepatocyte (−), CD3 (vessel+), GCDFP15 (+), and mammaglobin (+), with the diagnosis of right breast cancer recurrence (rT0N0M1, stage IV, lung and liver metastases). Magnetic resonance imaging (MRI) of the liver was also conducted and revealed multiple metastatic foci in the right segment of the liver. Brain, abdominal, and pelvic CT as well as a whole-body bone scan revealed no brain, bone, or any other metastases. The duration of disease-free survival of the patient was 5 years and 2 months.

### 2.2 Early Treatment and Partial Improvement of Recurrence

The patient received six cycles of advanced first-line TE regimen chemotherapy (T: docetaxel, 75 mg/m^2^; E: epirubicin, 60 mg/m^2^; intravenous injection, every 21 days as a cycle) from 23 June 2017, to 13 October 2017. Evaluation of the condition in the second, fourth, and sixth cycles revealed a stable disease. In October 2017, the patient was involved in a clinical trial involving adjuvant endocrine therapy. On 1 November 2017, the patient was administered anastrozole (1 mg/day, orally) and goserelin (3.6 mg/28 days, subcutaneously). The final evaluation on 30 August 2019, was a partial response based on the CT of the liver and lung, where no lesions were observed in the liver, and the lesions in the lung had significantly shrunk. However, a suspected larger intrahepatic focus was observed on abdominal CT on 21 November 2019, and on MRI on 25 November 2019, compared with the last evaluation (**Figures 2C–F**). CT of the lung did not show any progression of the pulmonary metastatic foci.

#### 2.2.1 Multiple Tumor Progression in Liver Metastasis of Advanced Breast Cancer

On 30 December 2019, the patient was involved in another randomized and double-blinded clinical trial and received treatment with a combination of SHR6390/placebo (150 mg/day, oral administration) and fulvestrant (250 mg/30 days, intramuscular injection). The evaluation of the patient’s disease progression revealed a stable disease during the period of administration. However, on 15 June 2020, liver lesion progression was confirmed by abdominal CT and MRI of the liver (**Figures 2G, H**). The patient received combined treatment with goserelin, exemestane, and palbociclib on 19 June 2020. On 11 December 2020, the evaluation revealed that the liver lesion was slightly larger than before based on abdominal CT and MRI of the liver (**Figures 2I, J**), and then the treatment was changed to letrozole and chidamide. Imaging showed that the liver lesion was larger than before on 26 February 2021 (**Figures 2K, L**), and eribulin monotherapy was administered on 11 March 2021. After 2 months, drug resistance developed, and abdominal computed tomography and MRI were performed (**Figures 2M, N**). In May 2021, local radiotherapy of the liver was performed 15 times at another hospital in Beijing. On 6 August 2021, an MRI of the liver revealed a space-occupying lesion on the upper segment of the right anterior lobe (range: 5.0 × 2.6 cm, **Figure 2O**). On 9 October 2021, an MRI of the liver showed that the lesion in the upper segment of the right anterior lobe of the liver was smaller than before, but new lesions were identified in the right posterior and left lateral lobes of the liver (**Figures 2P–R**). The treatment was changed to methotrexate on consensus by the multidisciplinary team and with the patient’s consent.

#### 2.2.2 The Patient’s Most Recent Status for Liver and Lung Metastases

**Figure 3:**
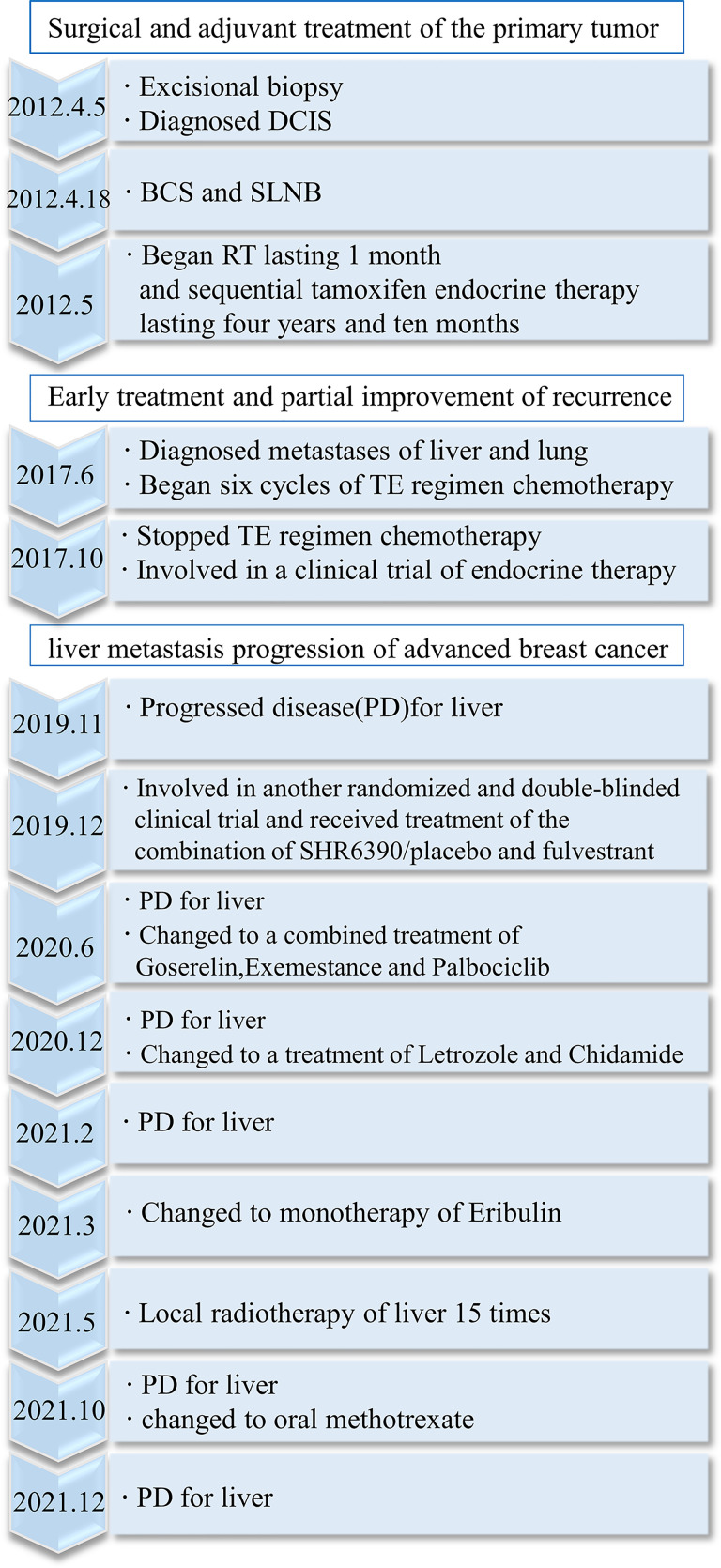
Timeline of the patient’s disease progression.

On 21 October 2021, the last CT scan of the lung showed that the lesions had apparently reduced (**Figure 2S**). On 15 December 2021, the last MRI of the liver showed that the range of the lesion on the right anterior lobe of the liver was larger, and perilesional enhancement on the right posterior and left lateral lobes of the liver was more obvious (**Figures 2T–V**). Overall, the metastatic focus of the lung was smaller, but there was a progression of liver metastasis (**Figure 3**).

## 3 Discussion

The World Health Organization Classification of Tumors of the Breast defines DCIS as a non-invasive precursor lesion that does not metastasize or cause death without progression to an invasive breast lesion. DCIS with microinvasion (≤0.1 cm in size) is defined as DCIS in the American Joint Committee on Cancer Staging Manual (3). As a non-invasive disease that is limited to the breast duct without invasion through the basal membrane (4), the potential of DCIS for DM has been ignored. Based on our research, it is uncommon to skip local recrudescence and discover DM, much less metastasis to more than one organ. There were no cases reported except for one (5) in Guangzhou, China, which involved multiple metastases of the bones and sigmoid colon following mastectomy for DCIS of the breast. The essential difference between the two cases is that the primary treatment in their case was modified radical mastectomy instead of partial mastectomy, and the pathological type of the tumor in their case was DCIS with microinvasion instead of pure DCIS (DCIS without any invasion). In terms of pathological characteristics and prognosis, DCIS with microinvasion is more similar to stage I breast carcinoma rather than pure DCIS and has a worse prognosis compared with that of pure DCIS (6–9). In that regard, DCIS with microinvasion is more likely to cause DM than pure DCIS. DM to multiple organs as the first event after primary treatment of pure DCIS has not been previously reported.

The distant organs where breast carcinoma preferentially metastasizes are the bone, liver, lung, and brain (10). Bone metastasis is most likely to occur in all types of breast carcinoma, particularly in the hormone receptor (HR)+/HER2+ type (11). Therefore, it is uncommon to develop liver and lung metastases rather than bone metastases, as what occurred in this case. In addition, pure DCIS caused multiple metastatic foci in the liver within 62 months, which was unexpected.

### 3.1 Reasons Underlying the Development of DMs in DCIS

At the cellular level, DCIS cells resemble invasive cancer cells (12). Research also demonstrated that by nature DCIS was truly a kind of cancer and possessed the ability to cause metastasis without invasion through the basement membrane (13). In several studies, approximately half of the patients who died from DCIS of the breast did not develop invasive breast cancer (13, 14). Besides, it has been stated that some patients diagnosed with pure DCIS without microinvasion still have positive lymph nodes (15–17). Other compelling evidence is that circulating tumor cells exist in the peripheral blood or bone marrow of patients diagnosed with pure DCIS (18, 19). These studies revealed the possibility of DCIS cells entering the circulating or lymphatic system before passing the basement membrane (20). Pathological research conducted by Gadre et al. indicated a possible mechanism (21). The researchers observed the presence of mucin in 36 of 41 ducts involved in DCIS (87.8%) and of mucin and vessels in 26 of 41 (63.4%). Variable amounts of CD68+ and VEGF+ macrophages identified in mucinous DCIS support the hypothesis that macrophages participate in the recruitment of facultative endothelial cells. In the four cases of mucinous DCIS without microinvasion, neovascularization was 100% present. This research also highlighted that DCIS is highly heterogeneous, and some subtypes have the potential to cause direct metastasis. Further research is required to facilitate detailed classification of DCIS according to histological appearance and molecular expression and to explore other underlying mechanisms.

### 3.2 Clinical Value of Serum Tumor Markers in Breast Cancer

Serum tumor markers are substances secreted by tumor cells or by the human body in response to the tumor and are elevated in the presence of malignant tumors (22). Carcinoembryonic antigen (CEA) and carbohydrate antigen (CA)153 are the most widely used serum tumor markers for breast cancer (23–25). CEA and CA153 have been recommended by the European Group on Tumor Markers to predict the prognosis and monitor the recurrence and therapeutic effects (26). CEA is extensively expressed in half of the breast cancers (27). CA153 has been proven to independently predict breast cancer recurrence and indicates the prognosis of advanced breast cancer (28). However, the combination of CEA and CA153 measurement does not possess satisfactory sensitivity in detecting metastasis of breast cancer (29), and the additional measurement of CA125, C-reactive protein (CRP), and other related biomarkers can improve the sensitivity of monitoring tumor progression (30). According to the study by Wang et al., CEA, CA153, and CA125 were detected to be higher in breast cancer patients with metastasis (31), and they were also related to tumor size, node status, and TNM stage (32, 33). CRP, as an inflammatory marker, was reported to be associated with poor prognosis in breast cancer (34–36) and increased significantly in the serum of patients with breast cancer metastasis (37). High serum levels of another inflammatory marker, β2-microglobulin (β2-MG), are also related to poor outcomes in metastatic breast cancer (36). Studies reported a protective relationship between serum alpha-fetoprotein levels and breast cancer (38, 39). However, according to a study by Zhao et al., alpha-fetoprotein exhibited no diagnostic significance in breast cancer nor did CA199 and CA724 (40).

In the present case, there were no abnormalities in the serum tumor marker levels, except CEA before metastasis was detected in May 2017. Subsequently, abnormal values of several other biomarkers were observed (**Table 1**). However, a slight increase in a single marker is not of great clinical significance. Other serum markers did not effectively predict metastasis, which is a reminder to clinicians not to be overly reliant on the diagnostic function of serum markers. During chemotherapy, the serum levels of CEA and CA153 gradually decreased, indicating the positive effect of chemotherapy.

**Table 1:** The levels of serum biomarkers during the case.

Date	CEA	CA125	CA153	β2-MG (serum)	AFP	NSE	CA724	CA199	Cyfra21-1	CRP	SCC	PCT
	0–4.3 ng/ml	0–35 U/ml	0–25 U/ml	0.7–1.8 mg/l	0–7 ng/ml	0–16.3 ng/ml	0–6.9 U/ml	0–27 U/ml	0–3.3 ng/ml	0–5 mg/ml	0–1.9 ng/ml	0–0.05 ng/ml
10 May 2013	2.35	24.74	6.65	NA	NA	NA	NA	NA	NA	NA	NA	NA
13 November 2013	1.87	25.40	7.94	NA	NA	NA	NA	NA	NA	NA	NA	NA
28 May 2014	2.97	23.00	7.57	NA	NA	NA	NA	NA	NA	NA	NA	NA
23 December 2014	3.45	20.82	7.48	NA	NA	NA	NA	NA	NA	NA	NA	NA
17 April 2015	3.48	12.38	6.78	NA	NA	NA	NA	NA	NA	NA	NA	NA
13 August 2015	2.96	13.76	6.24	NA	NA	NA	NA	NA	NA	NA	NA	NA
22 April 2016	3.50	12.91	7.10	NA	NA	NA	NA	NA	NA	NA	NA	NA
14 December 2016	4.07	28.25	14.51	NA	NA	NA	NA	NA	NA	NA	NA	NA
11 January 2017	4.34↑	NA	NA	NA	NA	NA	NA	NA	NA	NA	NA	NA
9 February 2017	4.51↑	14.48	17.90	1.45	4.17	13.43	6.71	<0.6	2.94	2.22	0.9	NA
1 June 2017	5.32↑	13.77	21.46	1.37	2.82	NA	7.09↑	<0.6	NA	2.70	NA	NA
3 July 2017	NA	NA	NA	1.58	NA	NA	NA	NA	NA	56.8↑	NA	0.09↑
15 July 2017	6.61↑	17.14	28.75↑	1.58	NA	NA	NA	NA	NA	4.35	NA	NA
9 August 2017	6.35↑	14.28	26.27↑	1.66	NA	NA	NA	NA	NA	11.3↑	NA	NA
16 September 2017	5.19↑	16.30	24.50	1.66	NA	NA	NA	NA	NA	5.00	NA	NA
31 October 2017	4.99↑	18.62	20.82	1.68	3.92	NA	NA	<0.6	NA	4.30	NA	NA
30 November 2017	4.57↑	14.55	11.98	NA	3.57	NA	NA	<0.6	NA	2.90	NA	NA
23 January 2018	4.40↑	11.90	6.70	1.9↑	NA	NA	NA	NA	NA	1.90	NA	NA
18 April 2018	4.39↑	12.61	6.60	1.68	NA	NA	NA	NA	NA	1.70	NA	NA
10 July 2018	4.43↑	12.83	5.97	1.91↑	NA	NA	NA	NA	NA	NA	NA	NA
29 September 2018	4.89↑	12.82	6.50	1.63	NA	NA	NA	NA	NA	NA	NA	NA
21 December 2018	4.77↑	11.46	6.49	1.55	2.40	NA	4.26	0.83	NA	NA	NA	NA
15 March 2019	4.62↑	13.21	6.38	1.49	2.88	NA	3.72	<0.6	NA	NA	NA	NA
6 June 2019	4.49↑	12.28	8.11	NA	2.69	NA	2.67	0.67	NA	<4	NA	NA
29 August 2019	4.87↑	12.76	8.50	NA	2.80	NA	2.36	1.04	NA	<4	NA	NA
21 November 2019	4.42↑	10.73	7.12	NA	2.59	NA	4.01	1.15	NA	<4	NA	NA
24 December 2019	4.51↑	12.60	8.61	NA	NA	NA	NA	NA	NA	NA	NA	NA
24 February 2020	4.35↑	12.90	9.78	NA	NA	NA	NA	NA	NA	NA	NA	NA
21 April 2020	4.23	12.50	9.57	NA	NA	NA	NA	NA	NA	NA	NA	NA
15 June 2020	4.06	12.20	11.10	NA	NA	NA	NA	NA	NA	NA	NA	NA
17 July 2020	5.17↑	10.20	11.80	NA	NA	NA	NA	NA	NA	NA	NA	NA
17 August 2020	4.92↑	9.47	13.20	NA	NA	NA	NA	NA	NA	NA	NA	NA
12 October 2020	3.95	12.20	13.30	NA	3.07	NA	NA	<2.00	NA	NA	NA	NA
11 December 2020	4.66↑	10.85	13.61	NA	NA	NA	NA	NA	NA	NA	NA	NA
26 February 2021	5.38↑	14.09	13.59	NA	2.41	NA	NA	1.13	NA	NA	NA	NA
21 April 2021	5.27↑	18.60	20.39	NA	3.81	NA	NA	1.27	NA	NA	NA	NA

NA, not accessible. ↑Abnormally elevated index.

### 3.3 The Risk Factors for DM in DCIS

The risk factors for developing DM in DCIS include younger age (≤40 years), lymph node metastasis, microinvasion, necrosis, little or no expression of hormone receptors, poor differentiation, previous or simultaneous invasive locoregional recurrence, positive HER2 expression, and high Ki-67 staining (>10%) (1, 5, 41, 42). In this case, there is no relevant risk factor except HER2 expression (3+) and high Ki-67 staining (25%). However, due to the rare rate of DM after DCIS, neither these risk factors nor the present treatment methods were statistically significant. Most distant metastases are detected after local recurrence (1). Therefore, further research is required in this regard.

### 3.4 Conclusions

This case demonstrates the poor potential outcome in pure DCIS, which has been underestimated for a long time. Further research should be conducted to determine the mechanisms, risk factors, and effective treatments for metastasis. Regular reexamination and monitoring are indispensable for patients after DCIS.

## Author Contributions

ZW, BC, and LZ: conceptualization. ZW and XZ: data and figure collection. ZW, XZ, and HR: writing—review and editing. All authors have reviewed and approved the final manuscript as submitted and agreed to be accountable for all aspects of the work. All authors contributed to the article and approved the submitted version.

## Conflict of Interest

The authors declare that the research was conducted in the absence of any commercial or financial relationships that could be construed as a potential conflict of interest.

## Publisher’s Note

All claims expressed in this article are solely those of the authors and do not necessarily represent those of their affiliated organizations, or those of the publisher, the editors and the reviewers. Any product that may be evaluated in this article, or claim that may be made by its manufacturer, is not guaranteed or endorsed by the publisher.

## Abbreviations

DCIS, ductal carcinoma *in situ*; DM, distant metastasis; BCS, breast-conserving surgery; DMs, distant metastases; IHC, immunohistochemistry; ER, estrogen receptor; PR, progesterone receptor; HER2, human epidermal growth factor receptor 2; CT, computed tomography; FISH, fluorescence *in situ* hybridization; MRI, magnetic resonance imaging; CEA, carcinoembryonic antigen; CA, carbohydrate antigen; CRP, C-reactive protein; β2-MG, β2-microglobulin; AFP, alpha-fetoprotein; NSE, neuron-specific enolase; SCC, squamous cell carcinoma antigen; PCT, procalcitonin.
